# Development and Application of an eDNA-Based MIRA-LFD Assay for the Detection of the Chinhai Spiny Newt

**DOI:** 10.3390/ani16203225

**Published:** 2026-10-14

**Authors:** Siling Ding, Ruonan Zhang, Xinyu Hu, Siyu Yang, Shimeng Dai, Aichun Xu, Feng Guan

**Affiliations:** 1 College of Life Sciences, China Jiliang University, Hangzhou 310018, China; 17764531132@163.com (S.D.);; 2 College of Science & Technology, Ningbo University, Ningbo 315300, China; 3 Department of Education, Michigan State University, East Lansing, MI 48824, USA

**Keywords:** wildlife surveys, eDNA, Chinhai spiny newt, MIRA-LFD, visual detection

## Abstract

The Chinhai spiny newt (*Echinotriton chinhaiensis*) is a rare amphibian species endemic to China. Its specialized habitat and small size make conventional field surveys and population monitoring not only time-consuming and labor-intensive, but also challenging. To address this challenge, we developed a visual detection method for habitat monitoring by integrating environmental DNA (eDNA) analysis with multi-enzyme isothermal rapid amplification (MIRA) and lateral flow dipstick (LFD) technologies. A specific mitochondrial gene fragment was selected as a target. MIRA primers and probes were designed and optimized through comparative sequence analysis with sympatric species, including crucian carp, bullfrogs, and polar fire shrimp. The assay conditions were subsequently refined by optimizing reaction temperature and test strip development time. Results indicate that using aquatic eDNA as a template, the optimized MIRA system incubated at 37 °C for 30 min, followed by a 20 min LFD development, produces clear and interpretable results. The assay demonstrated a DNA detection limit of 5.1 × 10^−4^ ng/μL, corresponding to a sensitivity higher than that of conventional PCR. The complete MIRA-LFD procedure can be performed within 50 min, and its identification results are consistent with those from high-throughput sequencing. This established method enables specific, efficient, and rapid detection of the Chinhai spiny newt in aquatic environments. It provides a practical technical tool for habitat surveys of this species, improving efficiency while reducing labor and resource demands. Furthermore, this approach offers a potential reference for habitat studies of other rare animal species.

## Simple Summary

The Chinhai spiny newt is a rare Chinese amphibian, but traditional field monitoring is difficult. In this study, we developed a rapid visual detection method by combining environmental DNA (eDNA) analysis with multi-enzyme isothermal rapid amplification (MIRA) and lateral flow dipstick (LFD) technologies. Targeting a specific mitochondrial gene, the assay was optimized for temperature and reaction time. Using aquatic eDNA, the method produces clear results within 50 min at 37 °C, with a detection limit of 5.1 × 10^−4^ ng/μL and sensitivity higher than conventional PCR. The results are consistent with high-throughput sequencing. This approach enables efficient, labor-saving habitat monitoring of the Chinhai spiny newt and provides a useful reference for studying other rare species.

## 1. Introduction

The Chinhai spiny newt (*Echinotriton chinhaiensis*) is an amphibian endemic to China with an extremely small wild population, but the current population size in the wild remains unknown [1,2]. It was previously reported in 2004 that the wild population was estimated at approximately 592 individuals. It is classified as Critically Endangered by the International Union for Conservation of Nature (IUCN) and is a first-class protected animal in China [2]. The species is primarily distributed in the Beilun District of Ningbo City, Zhejiang Province, China, where it inhabits broad-leaved evergreen forests and mixed forests at elevations between 200 and 500 m above sea level. To protect the Chinhai spiny newt, the Ningbo City government in Zhejiang Province established a nature reserve for this species in 2009 [3]. The reserve’s highest peak reaches 502.9 m, with an average slope of 15–25°, characteristic of coastal low-hill terrain. Each year from March to early to mid-May, the Chinhai spiny newt selects microhabitats with deciduous leaf litter and relatively low soil moisture (33.51 ± 1.87%) as spawning grounds, typically located under dead foliage or vegetation adjacent to semi-permanent still water puddles or puddles with very slow flow. After the egg sac hatches (under 18–22 °C about 20 day), the larvae enter the puddles and continue developing until they emerge onto land to begin their terrestrial life stage [3,4]. Given the spawning and hatching period of the Chinhai spiny newt, surveys for this species are typically conducted through direct observation on sunny afternoons [3,5], yet this species has rarely been sighted in the wild in recent years. Direct observation is labor-intensive, requires substantial resources, and is influenced by various environmental factors such as climate and microhabitat vegetation type, making field investigations highly challenging. Therefore, there is an urgent need to establish a straightforward survey and species identification method for the Chinhai spiny newt based on local environmental conditions and easily obtainable samples. As an important species for biodiversity conservation, the Chinhai spiny newt plays a significant role in maintaining ecosystem balance within its reserve, and as an indicator species it serves as an important warning signal for environmental changes and pollution. However, due to factors including habitat loss, climate change, and human activities, its populations have declined sharply and now face serious survival threats, the wild population may only number around 300 individuals [1]. The species also risks further decline from illegal capture and trade, a situation observed in the related Ryukyu spiny newt [6]. In summary, improving species identification methods and enhancing identification capability are essential both for field surveys and for verifying suspected remains of the Chinhai spiny newt, as well as for supporting efforts to combat illegal trade in other protected animals [7].

Environmental DNA (eDNA), defined as DNA released into the environment by organisms, holds significant potential for application in ecological research, species surveys, and biodiversity analysis [8,9,10,11,12,13,14]. Currently, eDNA-based techniques for animal surveys and species identification are developing rapidly. The primary methods applied include species-specific PCR [15,16,17,18], DNA sequencing (eDNA metabarcoding) [16,19,20,21,22,23], and other related approaches. eDNA metabarcoding is widely employed in amphibian monitoring, covering species such as *Lithobates catesbeianus*, *Ambystoma cingulatum*, and *Dicamptodon aterrimus* [24,25,26,27]. Compared to traditional field surveys, eDNA analysis offers considerable advantages in terms of convenience and reliability. Within the development of species-specific PCR identification methods, multienzyme isothermal rapid amplification (MIRA) has shown significant potential for diagnostic applications. Characterized by field compatibility, rapid amplification, high sensitivity, and visually interpretable results, MIRA has emerged as one of the most promising platforms in molecular diagnostics [28,29]. Furthermore, MIRA has been integrated with lateral flow dipstick (LFD) technology to enable rapid visual detection of amplification products [30,31,32]. It has been applied in fields such as authenticity identification of meats (beef, mutton, pork, chicken, and duck), identification of humans and common animals [33], and detection of specific species or pathogens including Feline parvovirus, Duck Hepatitis B Virus, goose astrovirus and *Bacillus cereus* [29,34,35,36,37]. In some pathogen detection assays, MIRA demonstrated superior reproducibility and stability, offering faster detection and simplified infrastructure requirements compared to real-time PCR, and even achieved the accuracy and sensitivity of qPCR [38,39]. Collectively, these studies provide a strong technical foundation for eDNA-based investigation and identification of the Chinhai spiny newt.

Based on the above conservation and survey requirements for *Echinotriton chinhaiensis* and the technical advantages of eDNA, this study aims to establish an accurate and simple detection method for field surveys to save labor and time.

## 2. Materials and Methods

### 2.1. Sample Collection and DNA Extraction

In April 2023, our research team collected Chinhai spiny newt specimens from the Chinhai spiny newt reserve in Beilun District, Ningbo, Zhejiang Province. The collection included four larvae and three adults, preliminarily identified as victims of cannibalism. After rinsing with purified water, the specimens were preserved in absolute ethanol for subsequent analysis. From August to September 2024, our team surveyed an area of approximately 5 km^2^ encompassing the reserve and adjacent homogeneous habitats to locate similar water bodies (≈1–20 m^2^). Using sterile plastic bottles, we collected approximately 2.0 L of subsurface water (from 10 cm below the surface) from the center of each water body, and these samples were transported back to the laboratory within 2 h after sampling and stored at 4 °C for later use. Filtration and DNA extraction were performed within 48 h. During this period, a total of 197 water samples were collected and transported to the laboratory, including five known positive-control water samples obtained from puddles within the protected area where the Chinhai spiny newts had been sighted during our field survey in April 2024.

Approximately 20 mg of subdermal muscle tissue was dissected from the Chinhai spiny newt specimens to serve as known positive controls. Genomic DNA was extracted using an Animal Tissue DNA Kit (No. 20240315, Hangzhou Xinjing Biotechnology Company, Hangzhou, China) according to the manufacturer’s protocol. For water samples, 200 mL aliquots were filtered through glass fiber microporous membranes (GF, 0.45 μm pore size, Φ47 mm, Shanghai Xingya Jinjing Company, Jinjing, China), and each membrane was cut into four pieces and stored at −80 °C until further use. One membrane piece was used for DNA isolation using the DNeasy Blood & Tissue Kit (QIAGEN, Cat. No. 69504, Hilden, Germany).

All extracted DNA was quantified using a NanoDrop 2000 spectrophotometer (Thermo Fisher Scientific, Waltham, MA, USA). Genomic DNA integrity was verified on 1.5% (*w*/*v*) agarose gel electrophoresis, and samples were stored at 4 °C for next use. DNA samples of six laboratory-preserved species, *Lithobates catesbeianus* (American bullfrog), *Carassius auratus* (crucian carp), *Sus scrofa domesticus* (pig), *Bos taurus* (cattle), *Ovis aries* (sheep), and *Neocaridina davidi* (cherry shrimp), were used as negative controls. These control specimens originated from waters within the reserve and part of these species co-occur with the Chinhai spiny newt in the same puddle; they were discovered during field surveys, sampled, and brought back to the laboratory. DNA from control samples of muscle tissues had been extracted using the aforementioned Animal Tissue DNA Kit and stored at −80 °C.

### 2.2. Water eDNA Species Identification by High-Throughput Sequencing

To confirm the animal species present in the collected water samples and to validate the species identification method developed in this study, five positive water samples (S1, S2, S3, S5, and S7) and five control samples (S9141, S9142, S91511, S91514, and S91517) were selected for high-throughput sequencing. The five positive samples were collected from puddles within the reserve where Chinhai spiny newts were known to be present, while the five control samples were taken from sites outside the reserve where Chinhai spiny newts had not been observed. Following eDNA extraction, high-throughput sequencing and species analysis were performed using 12S rRNA primers derived from the published literature [40], which are considered the most suitable for vertebrate species identification. The forward primer sequence was 5′-TAGAACAGGCTCCTCTAG-3′ (12SV05F), and the reverse primer sequence was 5′-TTAGATACCCCACTATGC-3′ (12SV05R). Primer design and analytical methodology followed established protocols [41] and Osathanunkul et al. [42]. eDNA amplifications were performed on a GeneAmp^®^ 9700 PCR System (Applied Biosystems, Foster City, CA, USA) in a total volume of 20 µL per reaction, containing 4 µL of 5× FastPfu Buffer (Mg^2+^ Plus) (TransGen Biotech Ltd., Beijing, China), 2 µL of 2.5 mmol/L dNTPs, 0.5 µL each of forward and reverse primers (10 µmol/L), 0.4 µL of FastPfu Polymerase (2.5 U/µL; TransGen Biotech Ltd., Beijing, China), 5 µL of DNA template (10–20 ng), and H_2_O to 20 µL.

PCR conditions included initial denaturation at 95 °C for 5 min; 32 cycles of denaturation at 95 °C for 30 s, annealing at 55 °C for 32 s, and extension at 72 °C for 40 s; followed by a final extension at 72 °C for 45 s and a hold at 4 °C. Triplicate PCRs were performed for each sample and mixed post-amplification to reduce potential PCR bias. PCR products were detected on a 2% agarose gel.

Positive PCR products were purified using the AxyPrep DNA Gel Extraction Kit (Axygen Biosciences, Union City, CA, USA), quantified with a QuantiFluor™-ST (Promega Corporation, Madison, WI, USA), and mixed at equimolar concentrations. Library construction was performed using the Illumina library builder kit (Vazyme Biotech Co., Ltd., Nanjing, China). Sequencing was carried out on the Illumina Novaseq 6000 SP (PE250, Illumina Inc., San Diego, CA, USA) platform by Shanghai Lingen Biotechnology Co., Ltd., Shanghai, China. Species identification was performed by comparing sequencing-derived reads (sequence similarity is greater than 98%) against two major reference databases: MitoFish (http://mitofish.aori.u-tokyo.ac.jp/download/, accessed on 20 April 2025) and NCBI NT (https://ftp.ncbi.nlm.nih.gov/blast/db/FASTA, accessed on 20 April 2025).

### 2.3. Confirmation of Chinhai Spiny Newt by DNA Barcoding

To verify the authenticity of the collected Chinhai spiny newt samples and ensure the accuracy of the target DNA sequence for this species, further species identification was performed by DNA sequencing. Mitochondrial DNA sequences were retrieved from the NCBI database for the following species: *Echinotriton chinhaiensis* (EU880315.1), *Lithobates catesbeianus* (NC_022696.1), *Carassius auratus* (NC_039257.1), *Neocaridina davidi* (NC_023823.1), *Sus scrofa domesticus* (NC_012095.1), *Bos taurus* (NC_006853.1), and *Ovis aries* (NC_001941.1). Homology alignment was conducted using the MegAlign software package (Lasergene v7.1, DNASTAR Inc., Madison, WI, USA). The mitochondrial 16S rRNA gene was selected as the diagnostic target, and species-specific primers were designed to amplify the Chinhai spiny newt DNA.

The primer sequences used were: Forward primer 16S-F: 5′-AGGCGATAAACCTAACGAGCC-3′; Reverse primer 16S-R: 5′-TCGTGATTGCCGAGTTCCTT-3′ (Expected amplicon size: 500 bp). The PCR conditions were as follows: initial denaturation at 95 °C for 5 min; 30 cycles of denaturation at 95 °C for 40 s, annealing at 61 °C for 40 s, and extension at 72 °C for 1 min; final extension at 72 °C for 7 min; followed by a hold at 4 °C.

Positive PCR products were subjected to bidirectional Sanger sequencing by Tsingke Biotechnology Co., Ltd., Hangzhou, China. Sequencing reads were aligned and assembled using Clustal X software (v2.1), followed by BLAST analysis against the GenBank database (https://blast.ncbi.nlm.nih.gov, accessed on 24 April 2025).

### 2.4. MIRA Primer Design and MIRA Reaction

The CytB gene (EU880315.1) was selected as the target for primer and probe design by comparing the mtDNA sequences of the Chinhai spiny newt with those of the negative control species, including amphibians, fish, as well as invertebrates such as shrimps and plankton in local nature reserves. Primers and probes were designed according to the MIRA kit instructions and taxon-specific primers selecting framework [43,44], with primer lengths generally ranging from 30 to 35 nt, probe lengths from 46 to 52 bp, and amplicon lengths from 150 to 300 bp [34]. Using Clone Manager software (Version 10, Sci Ed Software LLC, Cary, NC, USA), the following were designed: an upstream primer (MIRA-F), a downstream primer (MIRA-R1, with MIRA-R2 as an alternative), and a probe (MIRA-P1, with MIRA-P2 and MIRA-P3 as alternatives).

The 5′ end of the downstream primer was labeled with biotin. The probe was 5′-labeled with FAM, and a tetrahydrofuran (THF, dSpacer) moiety was incorporated approximately 23 nt from the 5′ end to serve as the recognition site for nfo (endonuclease IV). This THF site was positioned approximately 15 nt from the 3′ end. The 3′ terminus of the probe was modified with a C3 spacer group (Table 1).

To enhance probe specificity, locked nucleic acid (LNA) modifications were introduced at specific bases within the probe sequence, following reported methods [45,46]. All oligonucleotides were synthesized by General Biol (Anhui) Co., Ltd., (Chuzhou, China) and diluted to a working concentration of 10 µmol/L for subsequent use.

The MIRA assay was performed using a Recombinase Polymerase Amplification Kit (Weifang Amplfuture Biotechnology Co., Ltd., Weifang, China). A 50 μL reaction system was prepared according to the manufacturer’s instructions, containing 2 μL of each primer (using either the MIRA-F/R1 or MIRA-F/R2 combination), 29.4 μL of Buffer A, 2.5 μL of Buffer B, 5 μL of DNA template, and 9.1 μL of ddH_2_O. A negative control was included, consisting of pooled DNA from the six control species. Reactions were incubated at 37 °C for 30 min. Amplicons were then mixed 1:1 with a phenol:chloroform:isoamyl alcohol mixture (25:24:1, *v*/*v*/*v*) for protein extraction. Following centrifugation at 12,000× *g* for 5 min, the supernatants were analyzed by 2% (*w*/*v*) agarose gel electrophoresis. A comparative analysis was conducted to evaluate the specificity and amplification efficiency of the two primer sets (MIRA-F/R1 versus MIRA-F/R2).

For the selected primer pair (MIRA-F and MIRA-R1), temperature optimization was tested across five gradients (27, 32, 37, 42, and 47 °C) under standard MIRA-LFD conditions. Subsequently, reaction time optimization was performed at the optimal temperature, with incubation intervals of 10, 15, 20, 25, and 30 min. All experimental procedures were performed in triplicate.

### 2.5. MIRA-LFD Analysis and Primer-Probe Combination Selection

The MIRA-LFD reaction was performed using a Multi-enzyme Isothermal Amplification Colloidal Gold Kit (lateral flow dipstick type; Weifang Amplfuture Biotechnology Co., Ltd., Weifang, China). The 50 µL MIRA-LFD reaction mixture consisted of 29.4 µL of Buffer A, 9.1 µL of deionized H_2_O, 2.5 µL of Buffer B, and 2.0 µL each of the optimized primers MIRA-F and MIRA-R1, 3.0 μL of DNA template, and 2.0 μL of probe. Genomic DNA from the Chinhai spiny newt served as the positive control, while deionized water was used as a blank control. Colloidal gold test strips were used to visually interpret the results and identify the optimal probe. The 50 µL reaction mixture was incubated in a T100 PCR instrument (Bio-Rad Laboratories, Hercules, CA, USA) at a constant 37 °C for 30 min. After amplification, 10 µL of the product was diluted in 90 µL of deionized water. Then, 80 µL of the diluted mixture was loaded onto the sample well of a dual nucleic acid detection test strip (Sangon Biotech (Shanghai) Co., Ltd., Shanghai, China) for color-development and result observation. A tested sample was judged positive as long as a color change relative to the blank control was observed on the test strip. Results were visually interpreted after 5 min, with the C line serving as the control line and the T line as the test line.

### 2.6. MIRA-LFD Specificity Tests

To evaluate the specificity of the optimized MIRA primer-probe system developed in this study, testing was conducted under the above determined optimal reaction temperature and color development conditions. The assay used genomic DNA of the Chinhai spiny newt as the positive sample, a DNA mixture from six other species as the negative control, and ddH_2_O as the blank control. The species used as the negative control included *Rana catesbeiana*, *Carassius auratus*, *Neocaridina davidi*, *Sus scrofa domesticus*, cattle (*Bos taurus*), and sheep (*Ovis aries*). All experiments were repeated at least three times.

### 2.7. Determination of MIRA-LFD Sensitivity

The sensitivity of detection was determined using a genomic DNA serial dilution assay [34]. Five Chinhai spiny newt DNA samples were pooled in equal volumes and diluted to a concentration of 5.1 ng/µL. Using this diluted mixture as the template, a 10-fold serial dilution series was prepared (from 5.1 ng/µL to 5.1 fg/µL), with ddH_2_O serving as the blank control. The limits of detection of the MIRA-LFD assay were determined under optimized conditions: the MIRA reaction was conducted for 30 min, and results were recorded after a 20 min color development period. This detection was repeated independently six times. In parallel, conventional PCR amplification and MIRA were performed using the same primers MIRA-F and MIRA-R1. The products from both amplification methods were analyzed by agarose gel electrophoresis. The limits of detection for all three assays were then compared.

### 2.8. Water Sample Detection by MIRA-LFD and Conventional Species-Specific PCR

A total of 197 water eDNA samples were analyzed using the MIRA-LFD assay, with results observed and recorded after 20 min of color development. In parallel, the water eDNA samples were also analyzed by conventional species-specific PCR, following the method described in Section 2.3 and using the 16S-F/R primer set. Positive PCR products were submitted to Hangzhou Tsingke Biotechnology Co., Ltd. (Hangzhou, China) for bidirectional sequencing. The resulting sequences were analyzed using BLAST against the GenBank database. Finally, the numbers of positive samples detected by each method were tallied.

## 3. Results and Analysis

### 3.1. DNA Extraction and Species Identification for Chinhai Spiny Newt

Genomic DNA extracted from Chinhai spiny newt tissues exhibited concentrations ranging from 50.2 to 178.2 ng/μL (mean of 75.7 ± 48.3 ng/μL), with A_260_/A_280_ purity ratios between 1.8 and 2.0 (mean of 1.90 ± 0.11). The concentration and purity of the extracted DNA met the requirements for subsequent PCR analysis. Water eDNA concentrations ranged from 2.0 to 375.9 ng/μL (mean of 31.2 ± 46.6 ng/μL), with A_260_/A_280_ ratios from 1.4 to 2.3 (mean of 1.90 ± 0.18). These values were generally sufficient for PCR amplification. Although the complex mixture of pollutants and components in pond water samples can variably affect DNA extraction quality, the results indicated that the concentrations of the obtained extracts were adequate for different PCR amplifications.

**Figure 1:**
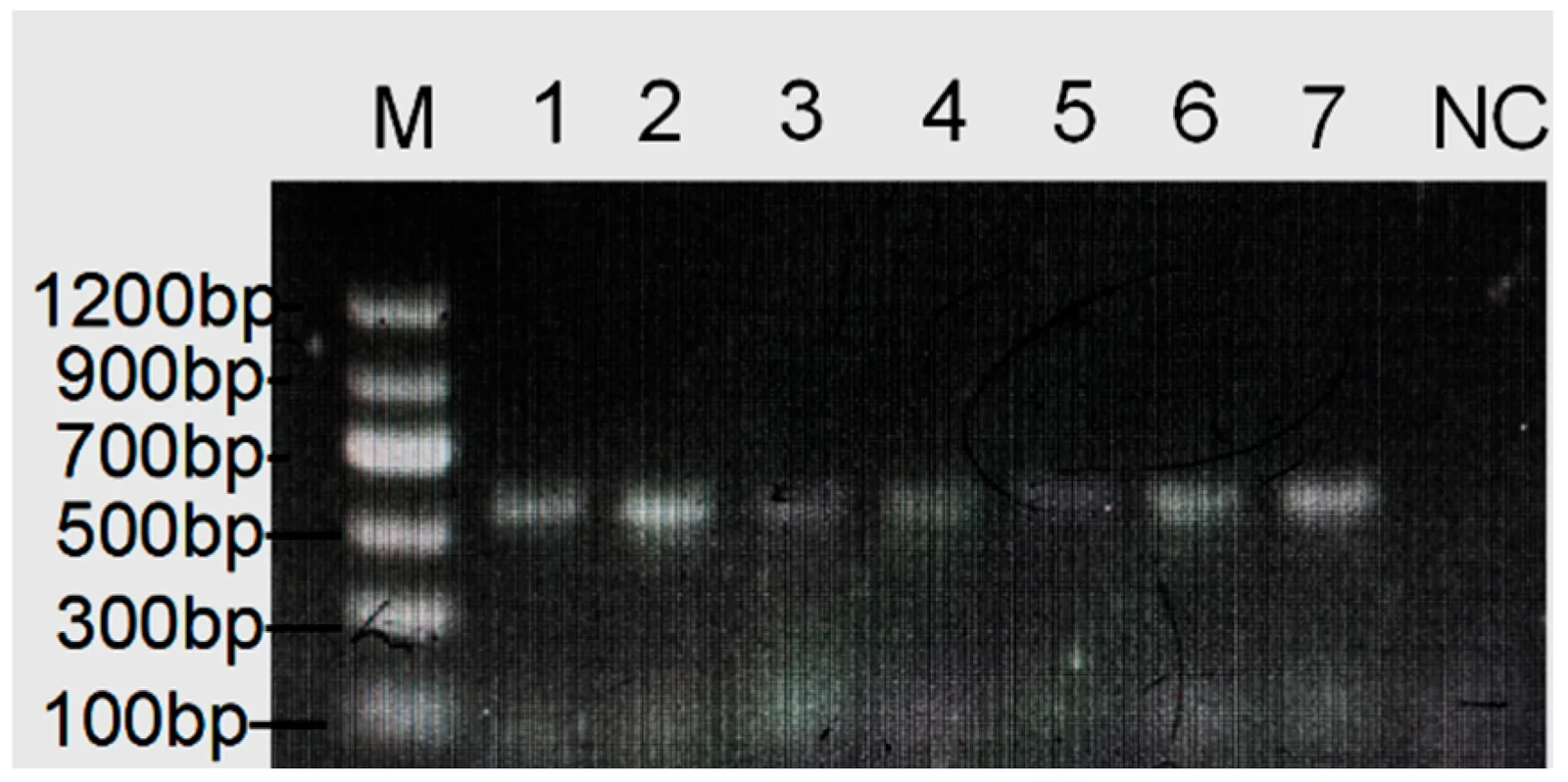
PCR amplification of the Chinhai spiny newt. Lane M: 1200 bp DNA marker. Lanes 1–7: Chinhai spiny newt samples. Lane NC: negative control.

DNA amplification of the Chinhai spiny newt using the primer pair 16S-F/R consistently yielded specific bands of approximately 500 bp on agarose gels (Figure 1), corresponding to the expected amplicon size. BLAST analysis of the sequenced products confirmed >98% identity with the Chinhai spiny newt reference sequence (EU880315.1), thereby verifying the species identity.

### 3.2. Species Identification in Water eDNA Based on High-Throughput Sequencing

**Figure 2:**
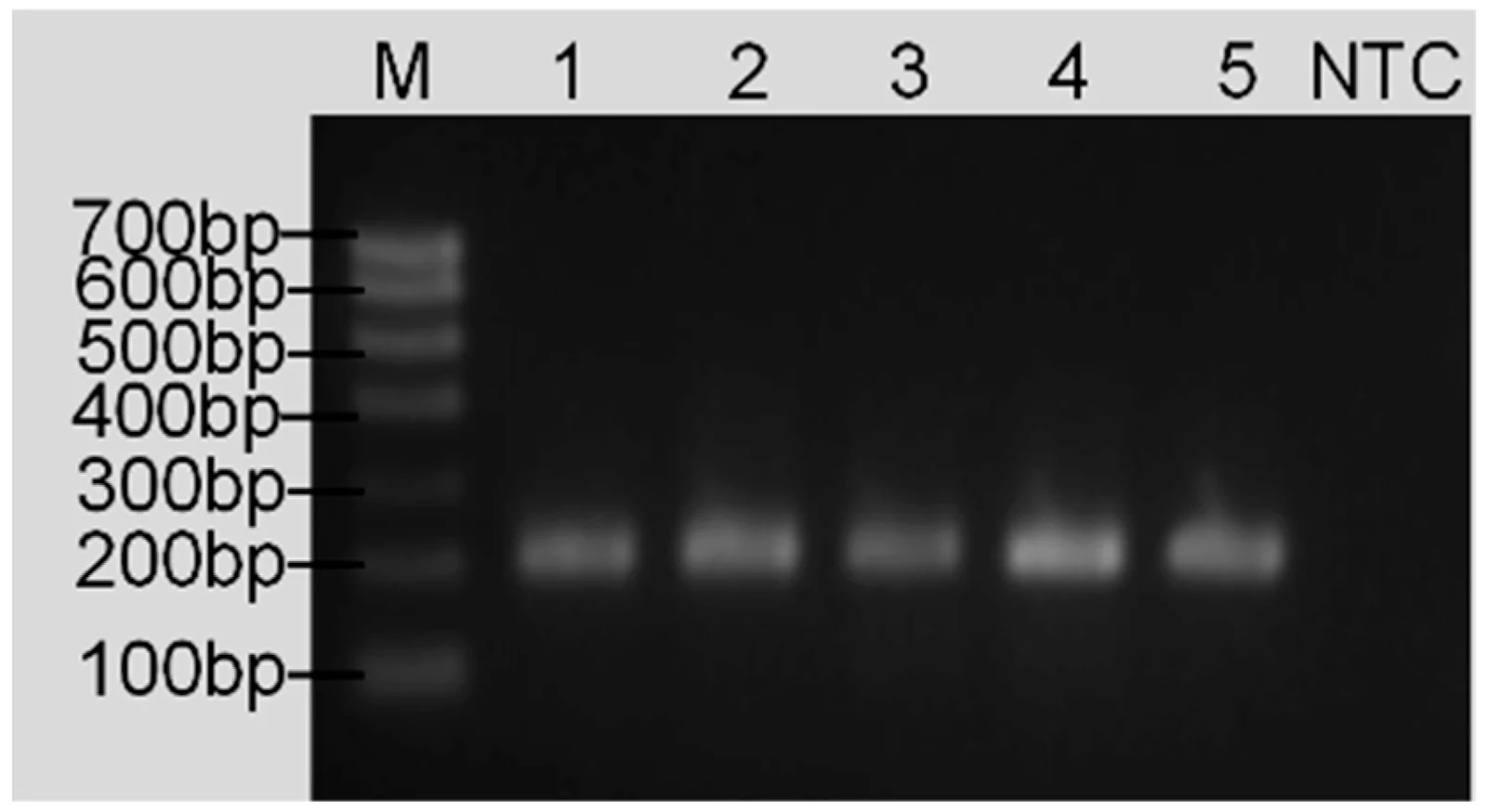
PCR amplification of water eDNA using 12S rRNA primers. Lane M: 1200 bp DNA marker. Lane 1: positive sample S1; Lane 2: positive sample S2; Lane 3: positive sample S3; Lane 4: control sample S9141; Lane 5: control sample S9142; Lane NTC: no-template control.

**Figure 3:**
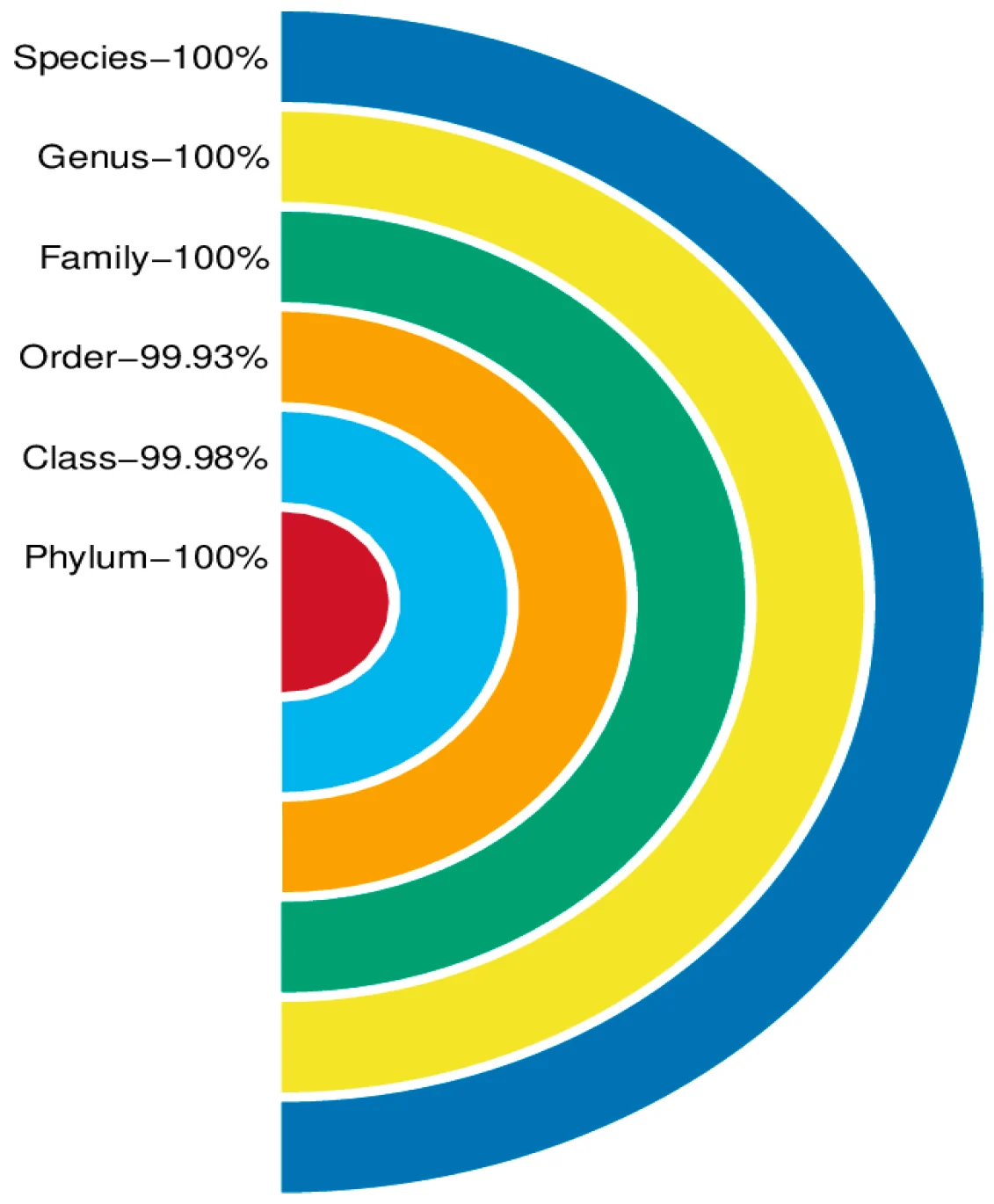
Statistical Pie Chart of Species Annotation Results for Water eDNA.

**Figure 4:**
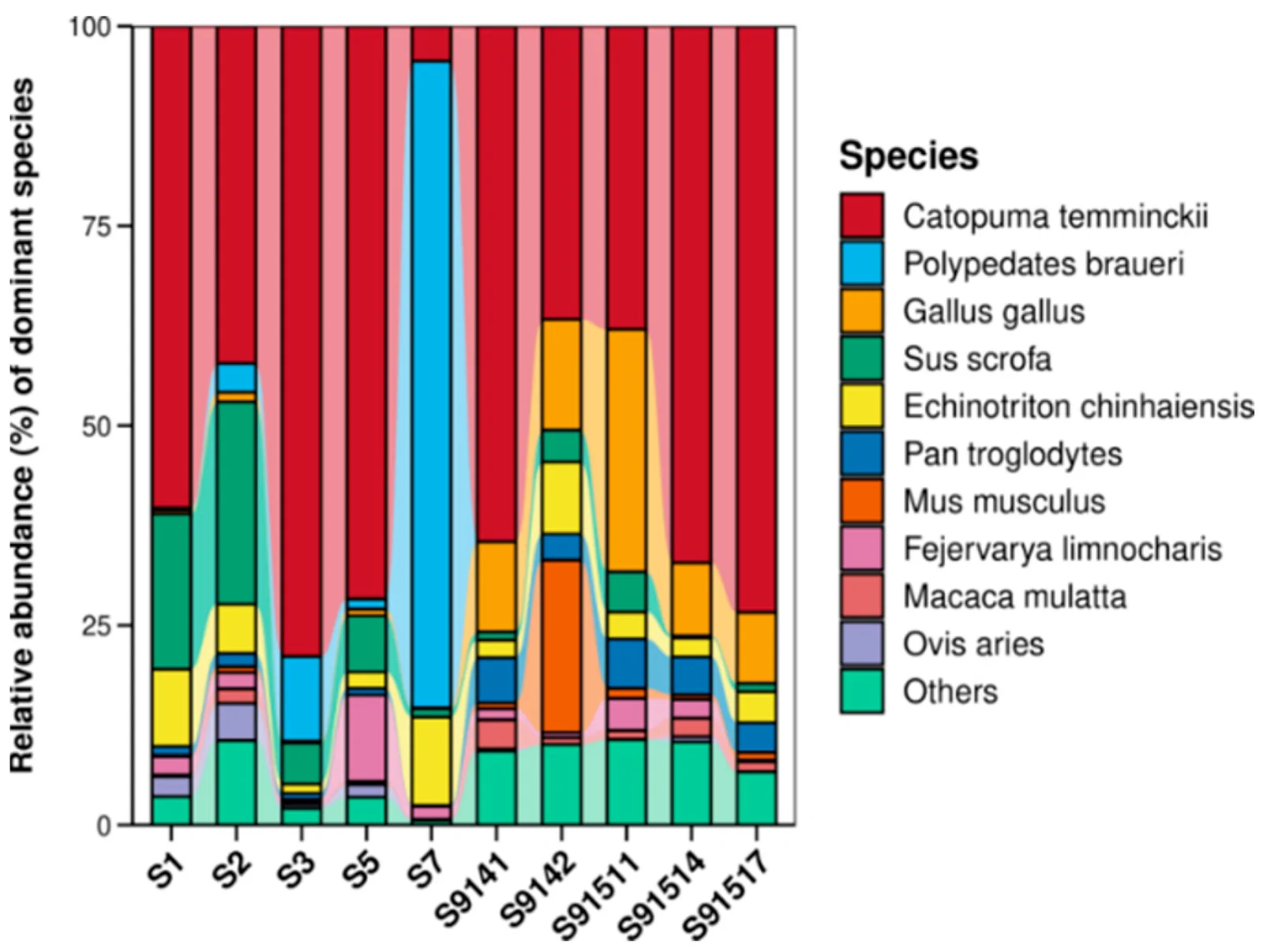
Accumulation diagram of 10 water eDNA samples at various taxonomic levels.

PCR amplifications were performed using 12S rRNA metabarcoding primers on eDNA from five positive water samples (collected within the reserve where Chinhai spiny newts were sighted) and five control samples (collected outside the reserve where Chinhai spiny newts are not observed). All ten amplicons produced fragments of the expected size on 2% agarose gels (Figure 2), confirming adequate eDNA quality and suitability for sequencing. Analysis of the high-throughput sequencing data yielded the following species annotation counts per sample: the five positive eDNA samples contained 54, 75, 49, 54, and 59 animal species, respectively, while the five control eDNA samples contained 96, 104, 84, 96, and 92 animal species, respectively. Species annotation was successful for all samples (100% success rate; Figure 3). A stacked bar plot (Figure 4) showed the Chinhai spiny newt among the ten most abundant species in all ten eDNA samples. Other identified species included *Catopuma temminckii* (Asian golden cat), *Rana splendida* (white-lipped tree frog), *Gallus gallus* (red junglefowl), *Sus scrofa* (wild boar), *Mus musculus* (house mouse), *Fejervarya limnocharis* (common marsh frog), *Ovis aries* (domestic sheep), *Bos taurus* (cattle), *Carassius auratus* (crucian carp), and *Neocaridina davidi* (red cherry shrimp). Although some taxonomically assigned species did not match the known local fauna of this region, this discrepancy likely arose from short read lengths, low read counts, and non-unique or low sequence identity matches against the reference database. These results demonstrate that eDNA high-throughput sequencing can effectively detect the presence of Chinhai spiny newts as well as other species in water samples.

### 3.3. Screening of MIRA Primers and Probes

**Figure 5:**
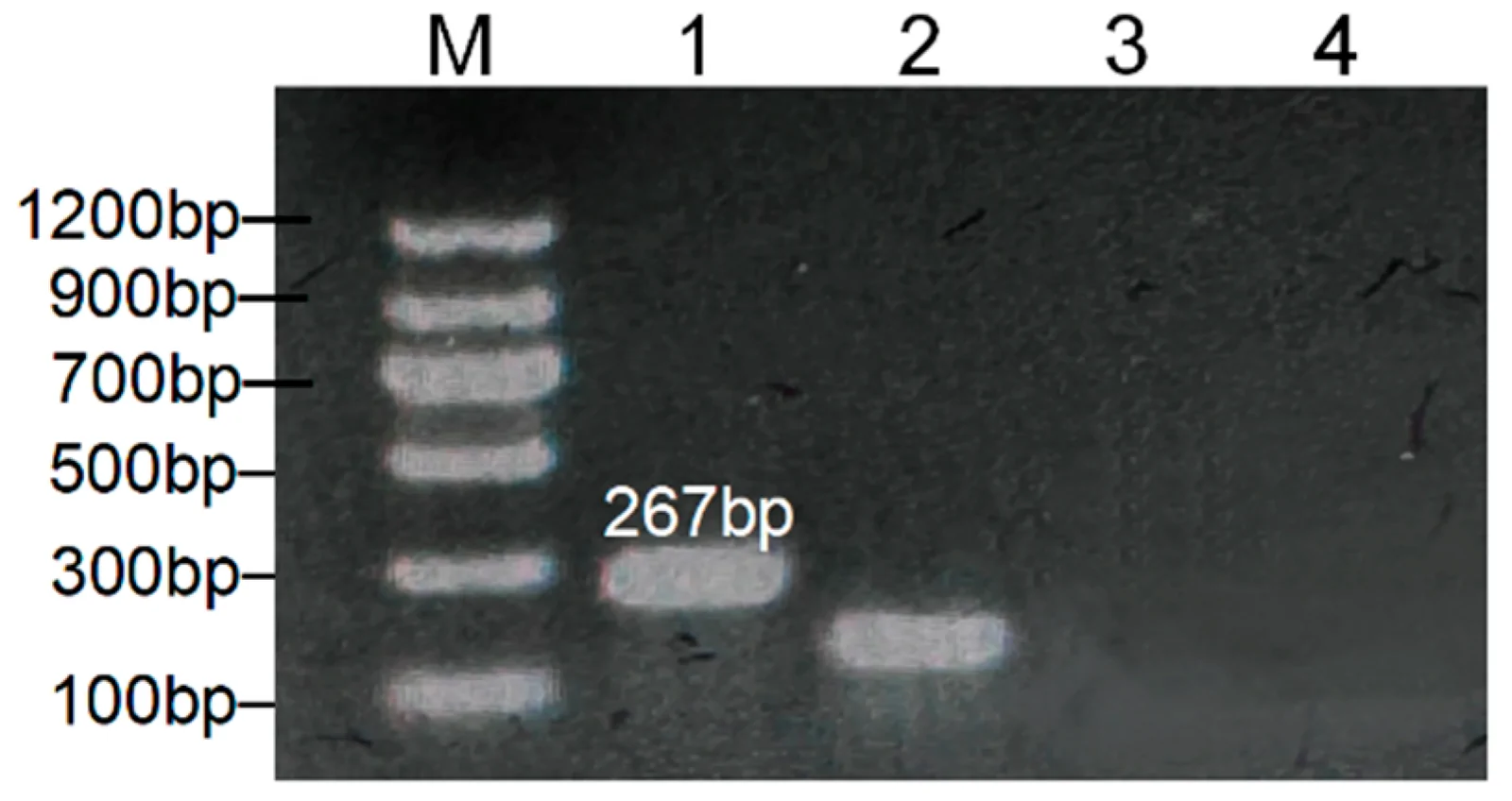
MIRA primers screening for the Chinhai spiny newt. Lane M: 1200 bp DNA marker; Lane 1: amplification product with primers MIRA-F/R1; Lane 2: product with primers MIRA-F/R2; Lanes 3 & 4: negative controls for MIRA-F/R1 and MIRA-F/R2, respectively.

**Figure 6:**
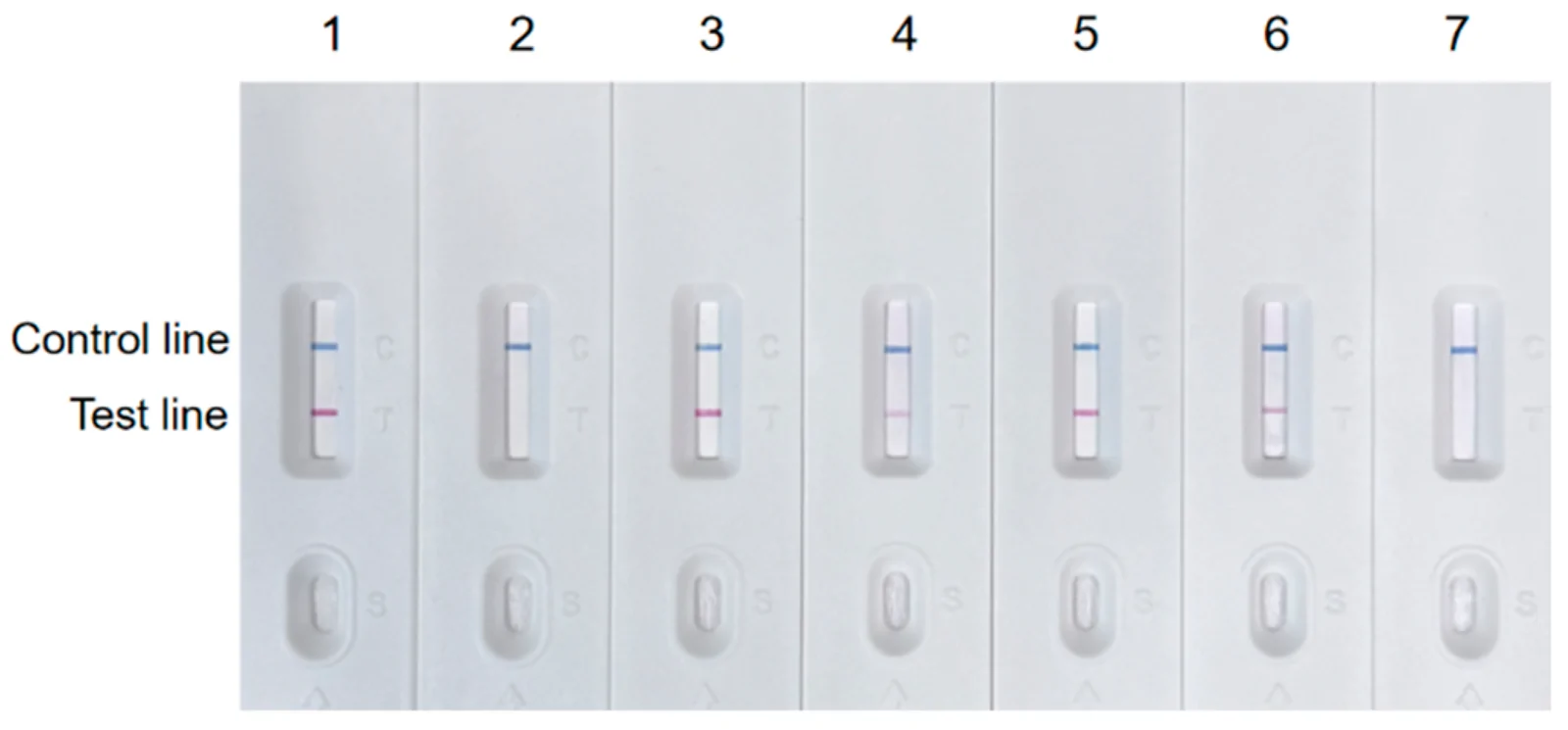
Screening of primer–probe combinations tested with Chinhai spiny newt DNA. Dipsticks 1 & 2: Probe MIRA-P1 (1) and negative control (2). Dipsticks 3 & 4: Probe MIRA-P2 (3) and negative control (4). Dipsticks 5 & 6: Probe MIRA-P3 (5) and negative control (6). Dipstick 7: ddH_2_O.

**Figure 7:**
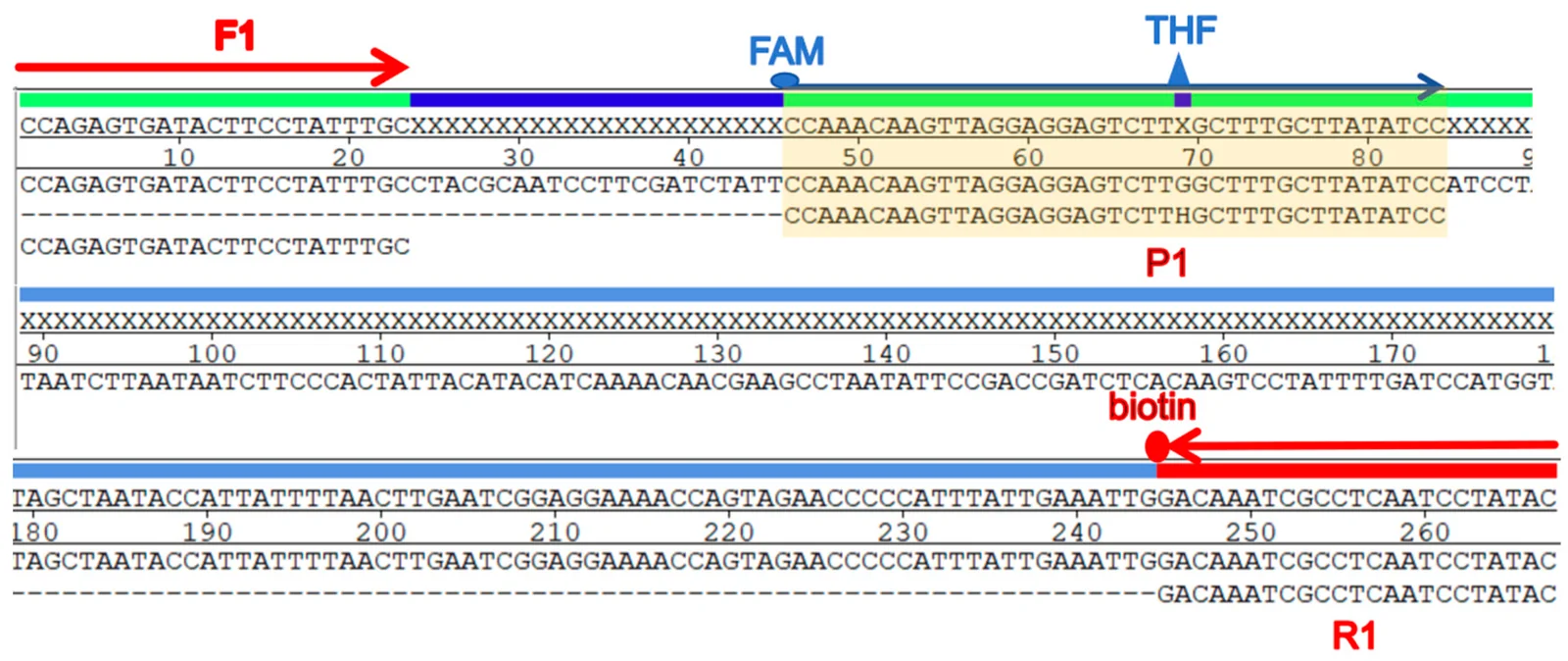
Design of MIRA-LFD primers and probe targeting the Chinhai spiny newt Cytb gene. F1, forward primer; R1, reverse primer; P1, probe.

Amplification results using two MIRA primer sets showed that both MIRA-F/R1 and MIRA-F/R2 specifically amplified the target sequences, producing fragments consistent with the expected sizes (Figure 5). According to MIRA design principles and negative control results, the MIRA-F/R1 primer set was selected as optimal. Specificity testing of three probes with the MIRA-F/R1 primers indicated that only the MIRA-P1 probe produced distinct positive signals for Chinhai spiny newt on lateral flow dipsticks, with no cross-reactivity observed in negative controls across replicates. In contrast, the MIRA-P2 and MIRA-P3 probes generated false-positive signals (Figure 6). These results demonstrated that the MIRA-F/R1 primer pair combined with the MIRA-P1 probe exhibited higher specificity than other primer-probe combinations. Consequently, this primer-probe detection system was established and selected for further optimization for Chinhai spiny newt species identification. The binding sites and locations of these oligonucleotides on the Cyt B gene sequence are illustrated in Figure 7.

**Figure 8:**
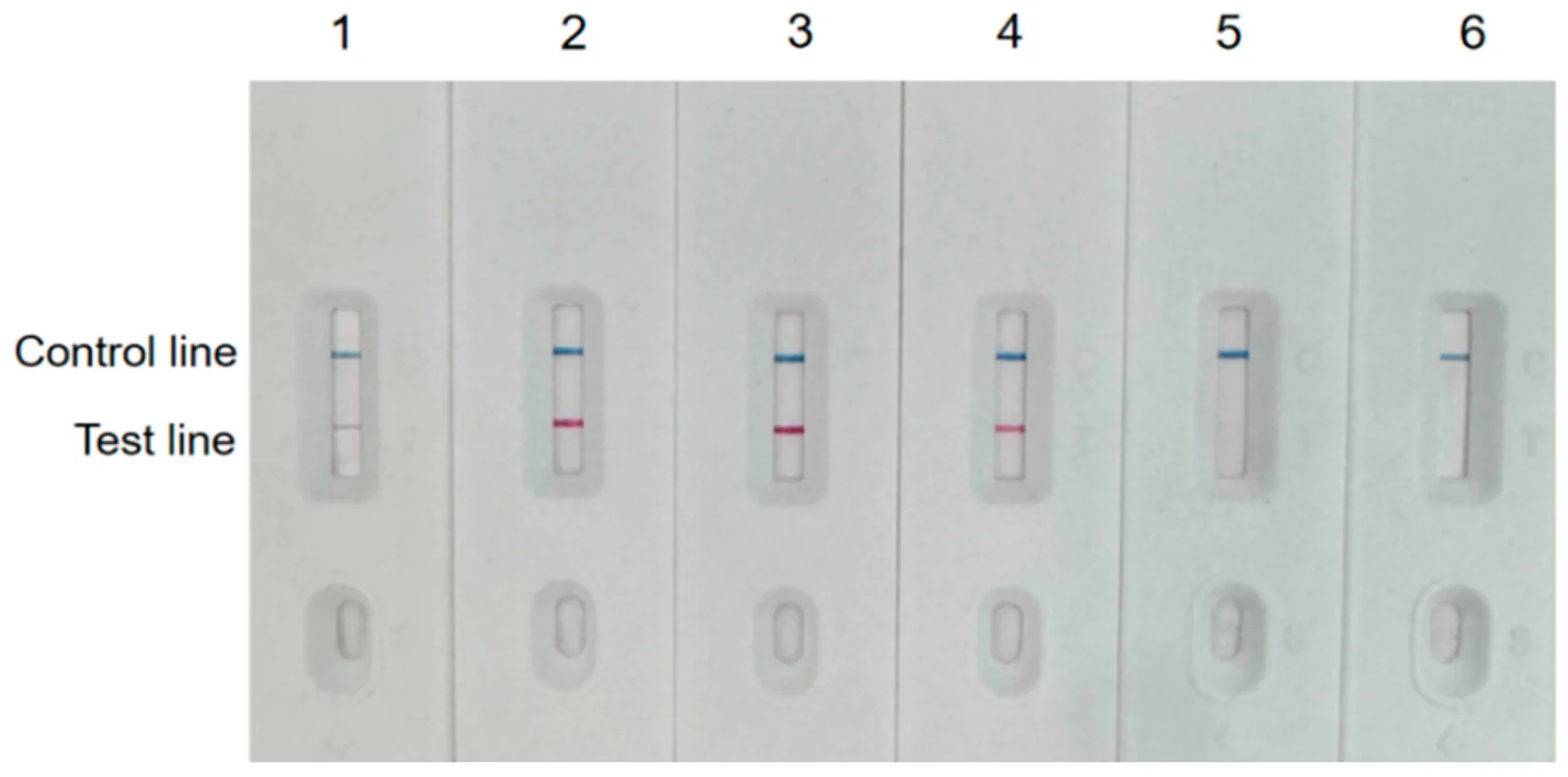
Optimization of MIRA-LFD reaction temperature (27–47 °C). Test strips 1–5 correspond to reaction temperatures of 27 °C, 32 °C, 37 °C, 42 °C, and 47 °C, respectively. Test strip 6: negative control (ddH_2_O).

**Figure 9:**
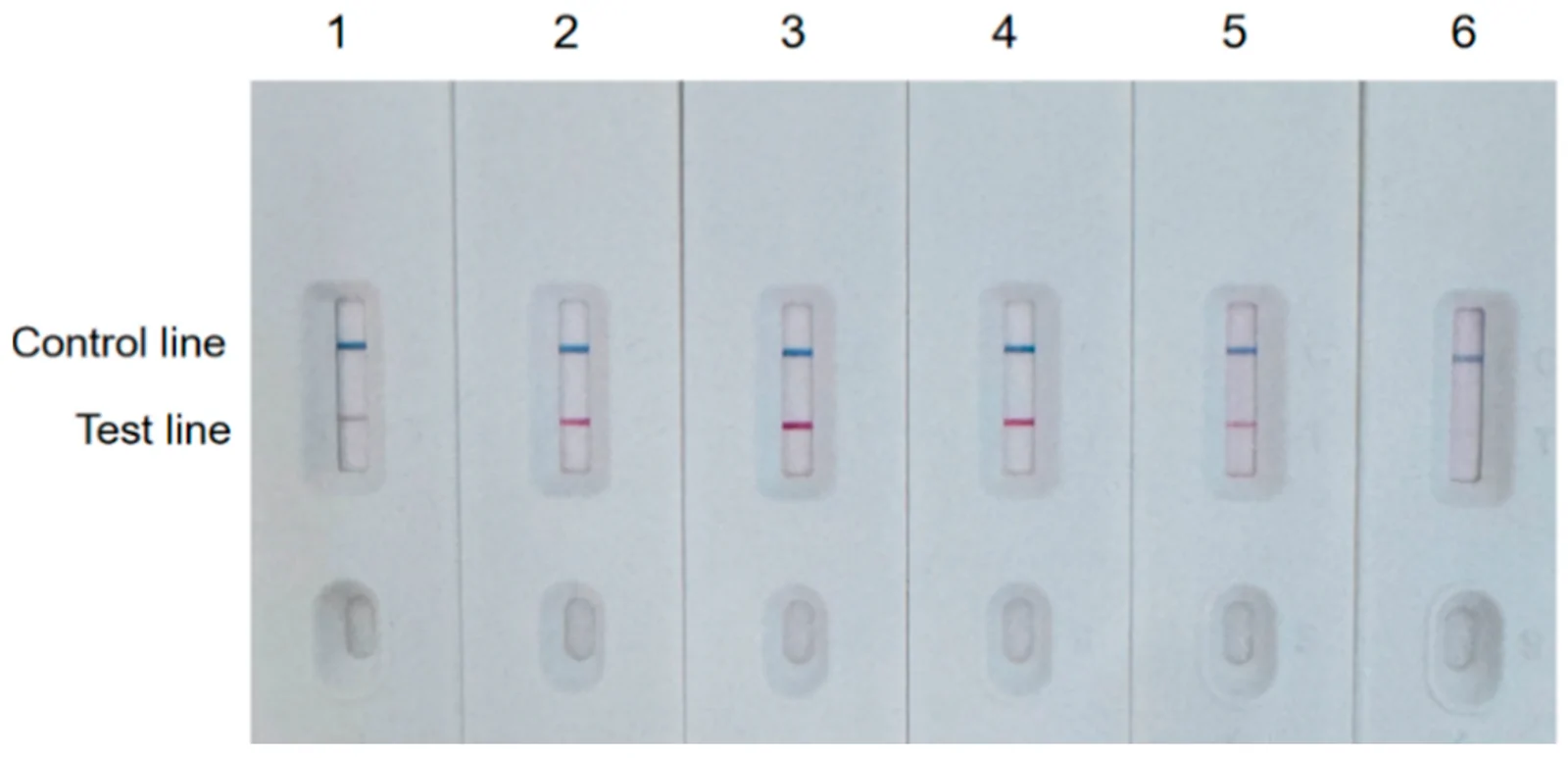
Time optimization of the MIRA-LFD color reaction at 37 °C. Test strips 1–5 correspond to color development times of 10, 15, 20, 25, and 30 min, respectively. Test strip 6: negative control (ddH_2_O).

Within the tested temperature range of 27 to 47 °C, LFD analysis of the MIRA products indicated that the signal obtained at 37 °C was the strongest and most specific (Figure 8). Subsequent amplification time optimization performed at 37 °C showed that test lines were visible across all tested intervals (10~30 min), with no discernible difference in color intensity between 20 and 25 min (Figure 9). Therefore, the following optimal conditions were established: amplification at 37 °C for 20 min, with a color development time of no less than 15 min, and 20~25 min being optimal.

### 3.4. Specificity of the MIRA-LFD

**Figure 10:**
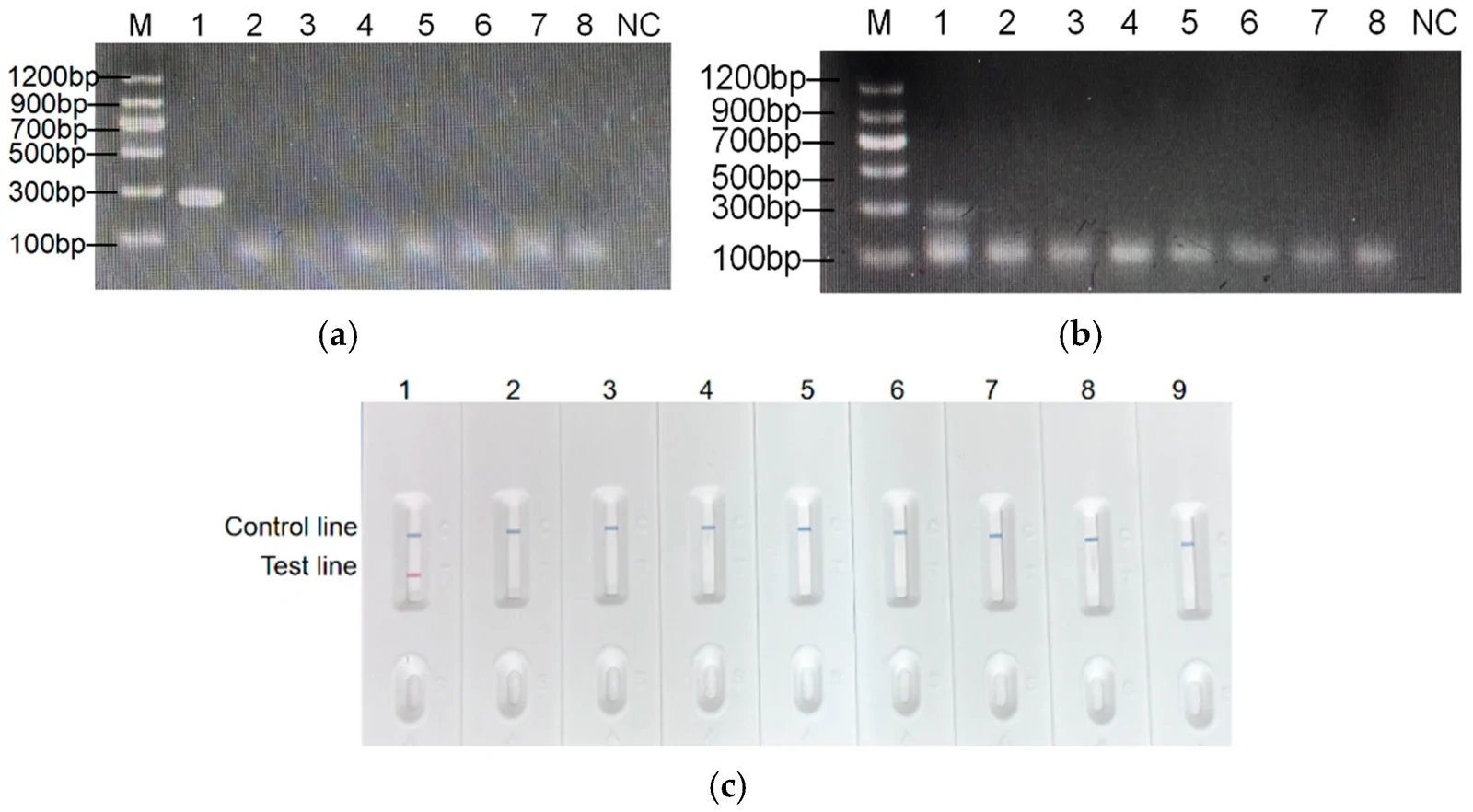
Specificity testing of the MIRA assay. Lane M: 1200 bp DNA marker in (**a**,**b**). (**a**) Conventional PCR using MIRA-F/R1 primers. (**b**) Isothermal MIRA amplification. (**c**) MIRA-LFD detection. Lane 1: *Echinotriton chinhaiensis* (Chinhai spiny newt); Lane 2: *Carassius auratus* (crucian carp); Lane 3: *Neocaridina davidi* (cherry shrimp); Lane 4: *Lithobates catesbeianus* (American bullfrog); Lane 5: *Bos taurus* (cattle); Lane 6: *Sus scrofa domesticus* (pig); Lane 7: *Ovis aries* (sheep); Lane 8: Negative control (no-template control, ddH_2_O); Lane NC: Blank control (no amplification reagents).

The specificity of the MIRA primers designed in this study was evaluated in two complementary assays. First, both conventional PCR and isothermal amplification were performed using the MIRA primers. Second, the amplification products were analyzed by agarose gel electrophoresis and LFD, respectively. The results demonstrated that the amplification products specifically identified the Chinhai spiny newt using both detection methods. Both the conventional PCR products (Figure 10a) and the isothermal MIRA amplification products (Figure 10b) displayed consistent specificity when analyzed by agarose gel electrophoresis. The band intensity for the conventional PCR product was stronger than that of isothermal MIRA, indicating the former has a higher amplification efficiency and product yield. The specificity observed by electrophoresis was consistent with the results from the LFD test strips (Figure 10c). Furthermore, the visual readout from the test strips was clearer and easier to interpret than that of the electrophoresis results, but it did not affect the specificity of the result. These findings confirm that the designed MIRA primer and probe combination exhibits high specificity and is suitable for detecting the Chinhai spiny newt in water eDNA samples.

### 3.5. Sensitivity of the MIRA-LFD

**Figure 11:**
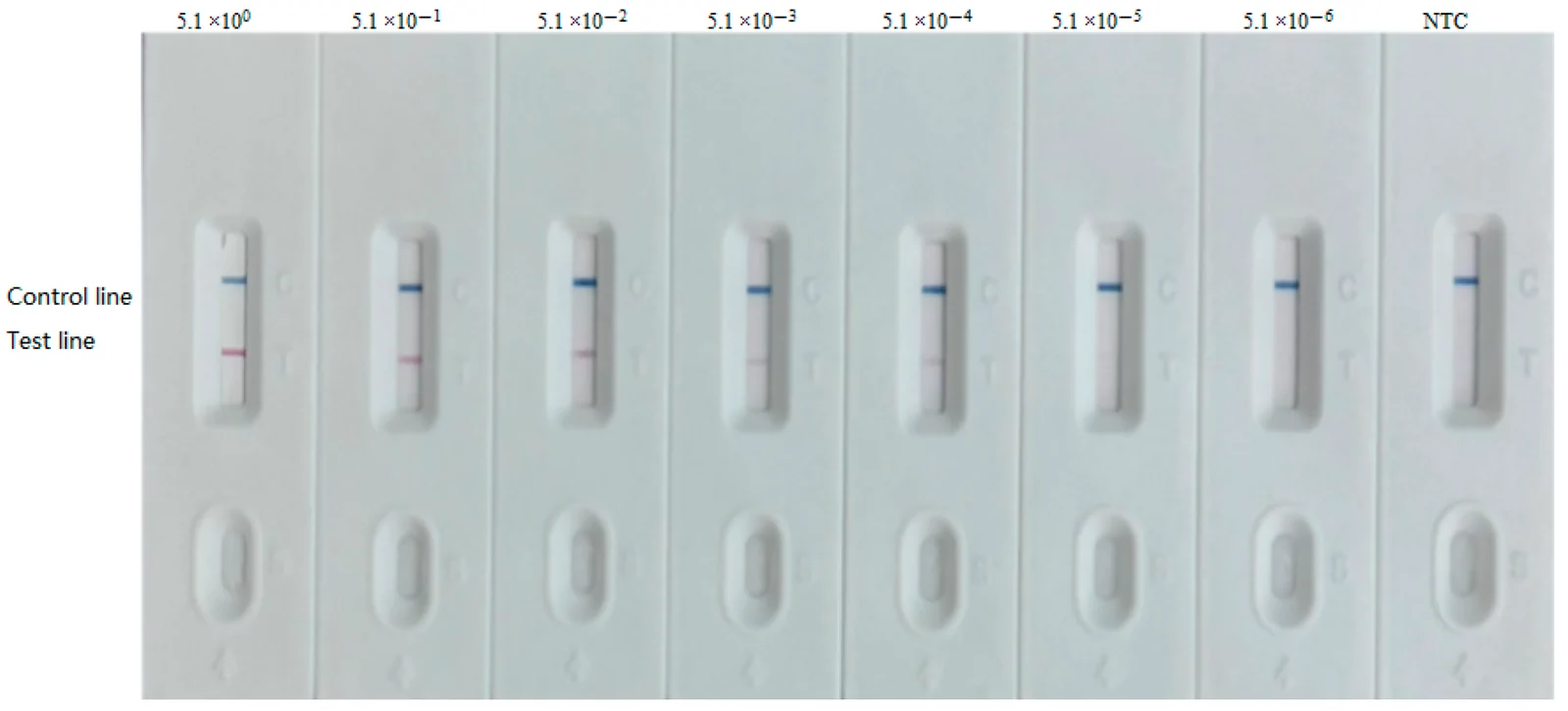
Determination of the detection limit by MIRA-LFD. The initial DNA concentration was 5.1 ng/μL. Reactions contained a 10-fold serial dilution series. NTC: no-template control (ddH_2_O).

**Figure 12:**
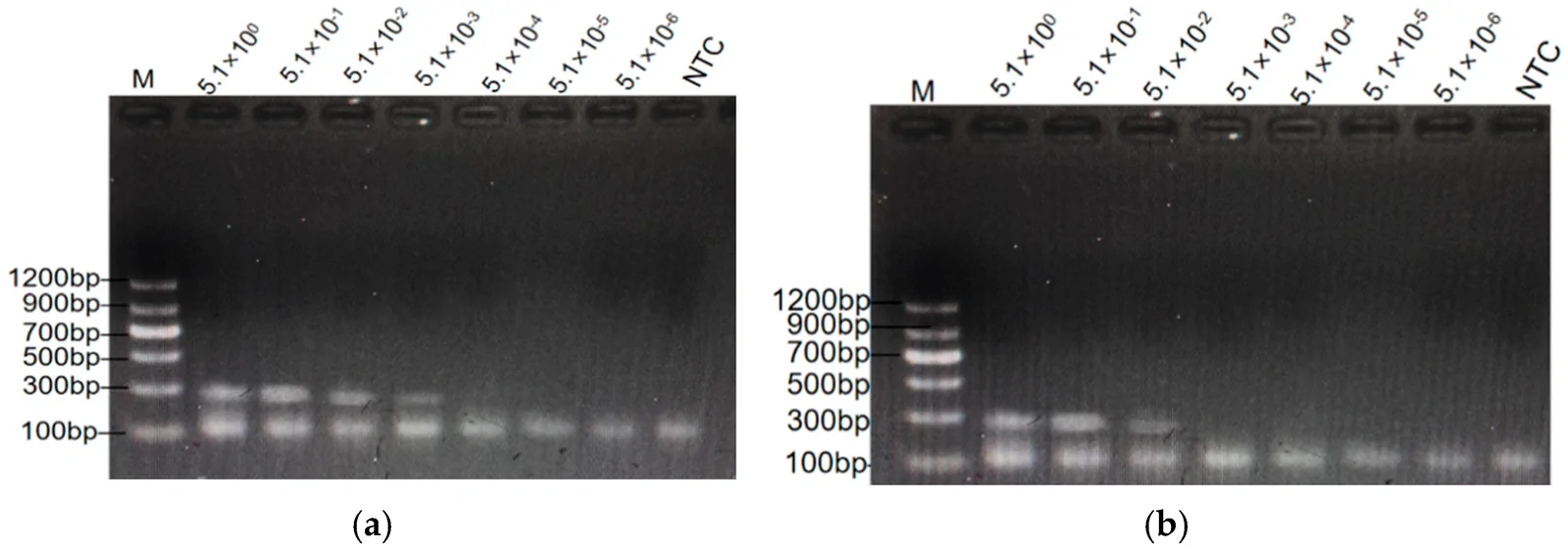
Determination of the detection limit by agarose gel electrophoresis. Lane M: 1200 bp DNA marker. (**a**) MIRA isothermal amplification products. (**b**) Conventional PCR amplification products using MIRA primers. The initial DNA template concentration was 5.1 ng/μL. Reactions contained a 10-fold serial dilution series. NTC: no-template control (ddH_2_O).

The sensitivity of the MIRA-LFD assay was evaluated using serial dilutions of pooled Chinhai spiny newt DNA sample. The results showed a progressive decrease in test line intensity with decreasing DNA concentration, with a faint but distinguishable line still observed at 5.1 × 10^−4^ ng/μL in PCR system (Figure 11). Comparative sensitivity analysis indicated that the detection limit for MIRA isothermal amplification was 5.1 × 10^−3^ ng/μL in PCR system (Figure 12a), while the detection limit for conventional PCR using the MIRA primers was 5.1 × 10^−2^ ng/μL in PCR system (Figure 12b). In comparison, the MIRA-LFD assay had a lower detection limit (5.1 × 10^−4^ ng/μL) than either conventional PCR or MIRA isothermal amplification with agarose gel detection. Consequently, the order of sensitivity was: MIRA-LFD > MIRA isothermal amplification > conventional PCR.

### 3.6. Water eDNA Detection Results

**Figure 13:**
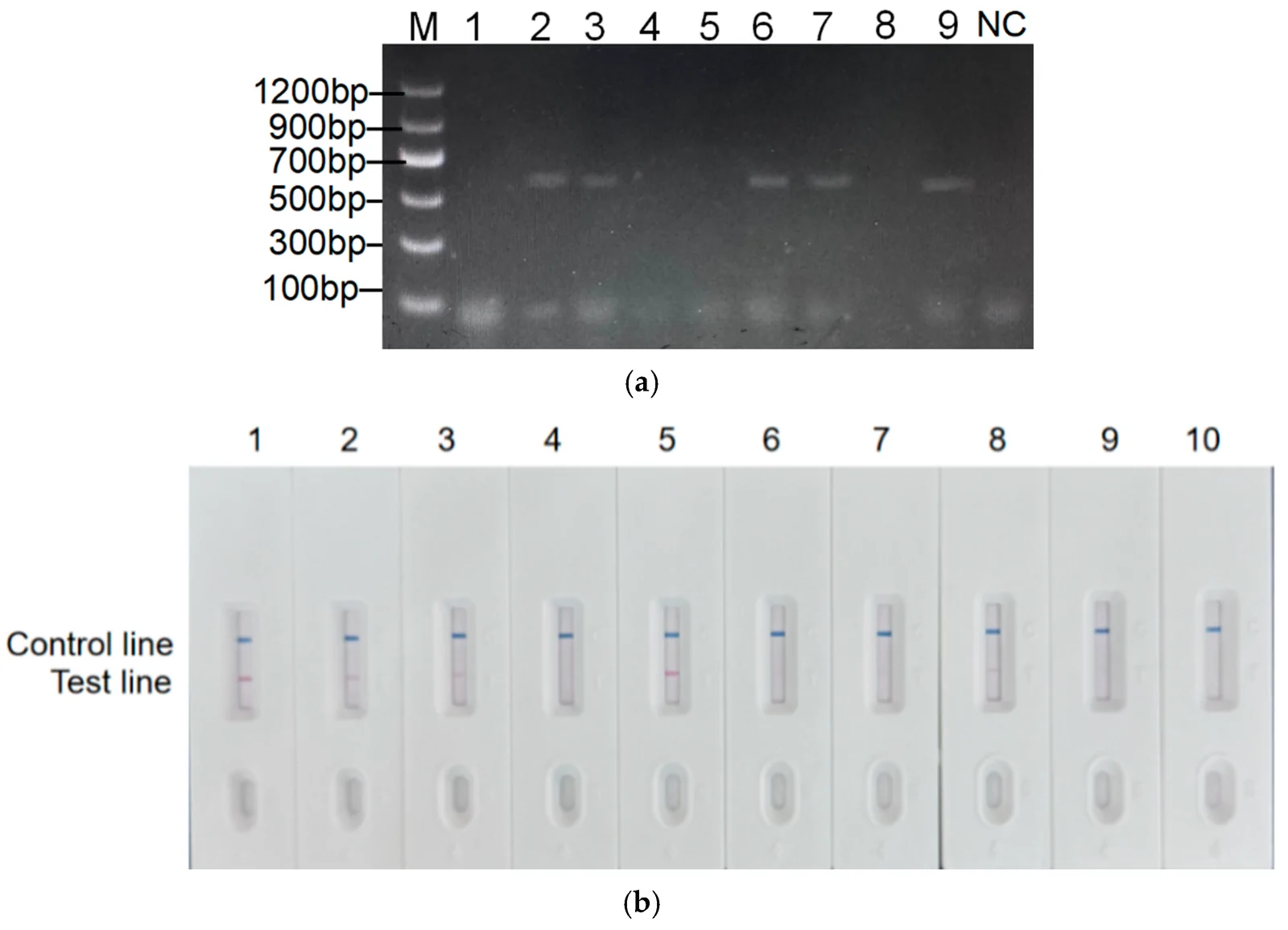
(**a**) Representative PCR results for water eDNA samples using 16S-F/R primers. Lane M: 1200 bp DNA marker. Lanes 1–9: water eDNA samples; Lane NC: negative control (ddH_2_O). (**b**) Representative MIRA-LFD detection results for water eDNA samples. Test strips 1–9: water eDNA samples; Test strip 10: negative control (ddH_2_O).

Conventional PCR was performed on 197 water eDNA samples using the 16S-F/R primers described in Section 2.3. Agarose gel electrophoresis showed that 51 samples produced positive amplification, yielding a product of approximately 500 bp consistent with the expected size (partial results shown in Figure 13a). Sanger sequencing of these PCR products followed by BLAST alignment against the NCBI database confirmed their identity as Chinhai spiny newt. Including the five known positive controls, a total of 51 samples were verified as positive, corresponding to a positivity rate of 25.89% (51/197). Partial results from MIRA-LFD detection are presented in Figure 13b. Distinct positive signals were observed for test strips 1, 2, 3, 5, and 8. Weak test line signals were visible for samples 6, 7, and 9. Samples showing weak signals were retested; those that remained positive were considered positive. The MIRA-LFD assay detected 84 positive samples, representing a detection rate of 42.64% (84/197, showed in Supplementary Materials). McNemar’s test was used to compare the detection positivity rates between the two methods, which has a significant difference (*p* < 0.05). These results indicate that the developed MIRA-LFD assay exhibits higher sensitivity than the PCR-sequencing method and can be used for effective detection of Chinhai spiny newts based on water eDNA. Furthermore, the results provide precise positioning for our future field surveys and this assay is amenable to adaptation for on-site field detection.

During the field survey in April 2026, we conducted on-site investigations at 84 eDNA-positive sampling sites of this study. Spawning was discovered at 40 puddles. This provides extremely accurate information for targeted field surveys and facilitates the conservation and research of Chinhai spiny newt.

## 4. Discussion

The Chinhai spiny newt is a rare, endemic urodele amphibian in China with a small population size. Determining the occurrence of this species is essential for conservation and requires sensitive and accurate detection methods. In recent years, our research team, along with other domestic and international researchers, has conducted field surveys and ecological studies on this species [3,47,48,49,50,51]. However, these studies have primarily focused on its distribution and life history, and the survey methods still largely rely on traditional line-transect and direct observation techniques [24]. This is also the most commonly used survey method for such small animals. There is limited research on specific species identification methods for the Chinhai spiny newt. Therefore, developing species identification protocols and eDNA-based detection technologies for this animal is of significant importance for its effective survey and conservation.

Modern biotechnology has provided crucial tools for animal species identification, including several DNA based methods, which offer methodological references for detecting the Chinhai spiny newt. However, a common limitation of DNA-based identification methods is their reliance on obtaining DNA directly from the target organism, which constrains their application for species like the Chinhai spiny newt where direct sampling is difficult. Fortunately, eDNA analysis has emerged as a tool that greatly simplifies this difficulty. Naturally, numerous successful cases are available for our reference. The integration of eDNA analysis with these sample types has significantly expanded the scope of species identification, facilitating surveys and monitoring of wildlife [19,20,52,53]. Consequently, our study developed a rapid and straightforward detection method for the Chinhai spiny newt using eDNA as the detection material, based on isothermal DNA amplification and LFD visualization. This approach enhances the accuracy of field surveys and offers the advantages of simple sampling and minimal time constraints. Based on the eDNA analysis results, targeted field surveys can be conducted to save time and labor costs.

Specificity is a critical parameter in DNA-based assays. To meet the specificity requirements of MIRA-LFD, our study selected the Cyt B gene sequence of the Chinhai spiny newt from GenBank as the target after comparative analysis. First, specific primers and probes were designed and screened. Their specificity was validated through high-throughput sequencing of eDNA, establishing an accurate MIRA-LFD detection method. Second, the results of the established MIRA-LFD method were compared with those from eDNA high-throughput sequencing, species-specific PCR followed by sequencing, and electrophoresis analysis of MIRA products. Third, locked nucleic acid-modified bases were introduced in the probe sequences, which can significantly improve the specificity of the probes [54,55]. These comparisons verified the method’s reliability and determined its limit of detection. Finally, both the MIRA-LFD method and specific PCR were applied to detect water eDNA samples to confirm the method’s practical utility. The optimized MIRA-LFD system demonstrated specific detection with no cross-reactivity to other co-occurring species in its habitat. The optimized MIRA-LFD system could amplify eDNA within 10–20 min at an optimal reaction temperature of 37 °C. The entire procedure was completed within 50 min, with a detection limit of 5.1 × 10^−4^ ng/μL. Compared with other reported MIRA applications, the optimal temperature used in this study is consistent with that used for identifying humans and seven common animal species [33], *Trichomonas vaginalis* [56], and Human T-cell lymphotropic virus-1 (HTLV-1) [57]. It is lower, however, than the 38 °C reported for duck hepatitis B virus [37] and the 42 °C for goose astrovirus detection [36]. Regarding amplification time, our results indicated that effective amplification could be achieved within 10–20 min. This range aligns with reported MIRA times for other targets, such as 10 min for duck hepatitis B virus [36], 12 min for goose astrovirus [36], 15 min for identifying humans and seven common animal species [33], 19 min for *Bacillus cereus* detection [34], and 20 min for HTLV-1 detection [56]. The amplification time in this study (10–20 min) is consistent with reports for similar MIRA assays. Considering the temperature parameters from these studies collectively, the data suggest a trend wherein higher reaction temperatures are associated with shorter amplification times.

Regarding sensitivity, the method developed here meets the detection requirements for eDNA samples. For comparison, reported limits of detection for other MIRA-based assays include: 10 pg (0.01 ng) for identifying humans and seven common animal species, a level equivalent to single-cell detection [33]; 12 fg/μL (1.2 × 10^−5^ ng/μL) for *Bacillus cereus* [34]; and a tenfold improvement over traditional nested PCR for feline parvovirus [29]. When combined with CRISPR/Cas12a for screening prostate cancer-related genes, MIRA has achieved a detection limit as low as 0.01 ng/L (1 × 10^−8^ ng/μL) [58], demonstrating substantially enhanced sensitivity. Our results also showed that the developed MIRA-LFD assay had higher sensitivity than conventional species-specific PCR. Although its sensitivity exceeds that reported for some similar technologies, the minimum eDNA concentration extracted from water samples in this study was 2.0 ng/μL, which is higher than that of the detection limit. Therefore, the MIRA-LFD assay fully meets the practical detection needs for aquatic eDNA. Furthermore, the assay can be performed at room temperature without expensive instrumentation. Following water sample filtration, it is suitable for rapid on-site detection. In addition, compared to eDNA high-throughput sequencing and common PCR, this method offers several advantages: isothermal amplification, visual interpretation of results without post-amplification processing, and high specificity.

In summary, this study established a visual detection method for the Chinhai spiny newt using eDNA and MIRA-LFD technology, which can be amenable to adaptation for field surveys of this species. Although we did not observe the Chinhai spiny newt in all positive water samples during the subsequent field surveys, this is partly attributable to the survey timing and sampling frequency. This assay significantly simplifies the survey and identification process while reducing demands on labor and material resources. It is possible that the species was present but not found. In future work, we will combine eDNA monitoring with field surveys to strengthen the conservation of these positive puddles.

Furthermore, we note that different water eDNA extraction methods and commercial kits can influence the sensitivity and accuracy of MIRA-LFD results. High-quality, high-concentration eDNA templates naturally enhance the assay’s sensitivity and success rate. The present method uses LFD visual signals to detect the Chinhai spiny newt specifically from eDNA samples. A current limitation is that this format does not support quantitative detection or high-throughput identification of multiple species simultaneously. However, multiplex LFD and multiplex MIRA-LFD platforms already exist [59], representing an important future direction for detection technology. In future, integrating eDNA analysis with such multiplex MIRA-LFD systems could potentially enable rapid, on-site identification of the specific or rare animal in field environments.

## 5. Conclusions

Our study established a visual detection method for the Chinhai spiny newt (*Echinotriton chinhaiensis*) based on water eDNA and MIRA-LFD technology. The method enables rapid and efficient monitoring of this species through water samples, exhibiting high specificity and relatively high sensitivity. It significantly facilitates field surveys and monitoring efforts for the Chinhai spiny newt and improves operational efficiency.

## Figures and Tables

**Table 1:** Sequences of MIRA primers and probes for Chinhai spiny newt.

Primer Name	Primer Sequence (5′-3′)	Product Size
MIRA-F	CCAGAGTGATACTTCCTATTTGC	
MIRA-R1 MIRA-R2	[Biotin]-GTATAGGATTGAGGCGATTTGTC [Biotin]-GACTTGTGAGATCGGTCGGAATA	267 bp 162 bp
MIRA-P1 MIRA-P2 MIRA-P3	FAM-CCAAACAAGTTAGGAGGAGTCTT[THF]GCTTTGCTTATATCC-[C3spacer] FAM-TCCAAACAAGTTAGGAGGAGTCT[THF]GGCTTTGCTTATATC-[C3spacer] FAM-TCCAAACAAGTTAGGAGGAGTCT[THF]GGCTTTGCTTATATC-[C3 spacer]	

Note: The bases within the boxes are locked nucleic acid-modified bases.

## Data Availability

The original contributions presented in this study are included in the article/Supplementary Materials. Further inquiries can be directed to the corresponding author.

## Supplementary Materials

The following supporting information can be downloaded at: https://www.mdpi.com/article/10.3390/ani16203225/s1, Table S1: PCR detection results of *Echinotriton chinhaiensis* based on eDNA samples.
