# Supplementary material for: Development and Application of an eDNA-Based MIRA-LFD Assay for the Detection of the Chinhai Spiny Newt
Source: Animals (Basel). 2026 Oct 14;16(20):3225. doi: 10.3390/ani16203225 (PMC13700770; doi:10.3390/ani16203225)
Supplement: Supplementary file 1 [file animals-16-03225-s001.zip › animals-4586886-supplementary.pdf]

Supplementary Materials

PCR detection results of *Echinotriton chinhaiensis* based on eDNA samples

Table S1. A Environmental DNA detection of water samples from water bodies within 5 km around the *Echinotriton chinhaiensis* Reserve (Taking the reserve as the center, the area outside the reserve is divided into grids at 1*1 kilometer intervals. The grids are numbered clockwise, and samples are collected and tested in each grid area)

No.	Sampling Date (M D)	Location	PCR Sequencing	similarity	*Echinotriton chinhaiensis*	MIRA-LFD
1(S1)	9.4	Reserve	False	/	/	Positive
2(S2)	9.4	Reserve	True	99.8%	Yes	Positive
3(S3)	9.4	Reserve	True	98.6%	Yse	Positive
4(S4)	9.4	Reserve	False	/	/	/
5(S5)	9.4	Reserve	True	99.79%	Yse	Positive
6(S6)	9.4	Reserve	True	100%	Yse	Positive
7(S7)	9.4	Reserve	False	/	/	Positive
8(9141)	9.13	Grid NO. 2	True	98.1%	Yse	Positive
9(9142)	9.13	Grid NO.2	True	96.7%	Yse	Positive
10(9143)	9.13	Grid NO. 2	False	/	/	/
11(9144)	9.13	Grid NO. 2	False	/	/	Positive
12(91511)	9.14	Grid NO. 2	False	/	/	Positive
13	9.14	Grid NO. 3	True	100%	Yse	Positive
14	9.14	Grid NO. 3	False	/	/	Positive
15	9.14	Grid NO. 3	True	100%	Yse	Positive
16	9.14	Grid NO. 3	True	100%	Yse	Positive
17	9.14	Grid NO. 3	True	99.76%	Yse	Positive
18	9.14	Grid NO. 3	False	/	/	Positive
19	9.14	Grid NO. 3	True	100%	Yse	Positive

20	9.14	Grid NO. 3	False	/	/	/
21	9.18	Grid NO. 4	False	/	/	/
22	9.18	Grid NO. 4	True	98.33%	Yse	Positive
23	9.18	Grid NO. 4	False	/	/	Positive
24	9.18	Grid NO. 4	True	97.77%	Yse	Positive
25	9.18	Grid NO. 4	True	99.59%	Yse	Positive
26	9.18	Grid NO. 4	False	/	/	/
27	9.18	Grid NO. 4	False	/	/	Positive
28	9.18	Grid NO. 4	False	/	/	/
29	9.18	Grid NO. 4	False	/	/	Positive
30	9.18	Grid NO. 4	True	99.8%	Yse	Positive
31	9.18	Grid NO. 4	False	/	/	/
32	9.18	Grid NO. 4	False	/	/	Positive
33	9.20	Grid NO. 5	False	/	/	Positive
34	9.20	Grid NO. 5	False	/	/	/
35	9.20	Grid NO. 5	False	/	/	Positive
36	9.20	Grid NO. 5	True	100%	Yse	Positive
37	9.21	Grid NO. 5	False	/	/	Positive
38	9.21	Grid NO. 5	True	98.7%	Yse	Positive
39	9.21	Grid NO. 5	True	99.6%	Yse	Positive
40	9.21	Grid NO. 5	False	/	/	Positive
41	9.21	Grid NO. 5	True	99.79%	Yse	Positive
42	9.23	Grid NO. 6	True	99.8%	Yse	Positive
43	9.23	Grid NO. 6	True	100%	Yse	Positive
44	9.23	Grid NO. 6	True	100%	Yse	Positive
45	9.23	Grid NO. 6	True	100%	Yse	Positive
46	9.23	Grid NO. 6	True	100%	Yse	Positive
47	9.23	Grid NO. 6	True	100%	Yse	Positive

48	9.23	Grid NO. 6	True	100%	Yse	Positive
49	9.23	Grid NO. 6	True	100%	Yse	Positive
50	9.23	Grid NO. 6	False	/	/	Positive
51	9.23	Grid NO. 6	True	100%	Yse	Positive
52	9.23	Grid NO. 6	True	100%	Yse	Positive
53	9.23	Grid NO. 6	True	100%	Yse	Positive
54	9.23	Grid NO. 6	True	100%	Yse	Positive
55	9.23	Grid NO. 6	True	100%	Yse	Positive
56	9.23	Grid NO. 6	True	100%	Yse	Positive
57	9.23	Grid NO. 6	True	100%	Yse	Positive
58	9.23	Grid NO. 6	False	/	/	Positive
59	9.23	Grid NO. 6	False	/	/	Positive
60	9.24	Grid NO. 6	False	/	/	Positive
61	9.24	Grid NO. 7	True	100%	Yse	Positive
62	9.24	Grid NO. 7	True	100%	Yse	Positive
63	9.24	Grid NO. 7	True	100%	Yse	Positive
64	9.24	Grid NO. 7	True	100%	Yse	Positive
65	9.26	Grid NO. 8	True	100%	Yse	Positive
66	9.26	Grid NO. 8	False	/	/	Positive
67	9.26	Grid NO. 8	False	/	/	Positive
68	9.26	Grid NO. 8	False	/	/	Positive
69	9.26	Grid NO. 8	False	/	/	Positive
70	9.26	Grid NO. 8	True	100%	Yse	Positive
71	9.29	Grid NO. 9	False	/	/	/
72	9.29	Grid NO. 9	False	/	/	/
73	9.29	Grid NO. 9	False	/	/	Positive
74	9.29	Grid NO. 9	False	/	/	/
75	9.29	Grid NO. 9	False	/	/	/
76	9.29	Grid NO. 9	False	/	/	/
77	9.29	Grid NO. 9	False	/	/	/

78	9.29	Grid NO. 9	False	/	/	Positive
79	9.29	Grid NO. 9	False	/	/	/
80	9.29	Grid NO. 9	False	/	/	/
81	9.29	Grid NO. 9	False	/	/	/
82	9.29	Grid NO. 9	False	/	/	/
83	9.29	Grid NO. 9	False	/	/	Positive
84	10.4	Grid NO. 10	True	100%	Yse	Positive
85	10.4	Grid NO. 10	False	/	/	/
86	10.4	Grid NO. 10	False	/	/	/
87	10.4	Grid NO. 10	True	99.4%	Yse	Positive
88	10.4	Grid NO. 10	False	/	/	/
89	10.4	Grid NO. 10	False	/	/	/
90	10.4	Grid NO. 10	True	100%	Yse	Positive
91	10.4	Grid NO. 10	False	/	/	/
92	10.9	Grid NO. 11	False	/	/	Positive
93	10.9	Grid NO. 11	False	/	/	Positive
94	10.9	Grid NO. 11	False	/	/	Positive
95	10.9	Grid NO. 11	True	100%	Yse	Positive
96	10.9	Grid NO. 11	False	/	/	Positive
97	10.9	Grid NO. 11	True	99.8%	Yse	Positive
98	10.9	Grid NO. 11	True	100%	Yse	Positive
99	10.9	Grid NO. 11	True	100%	Yse	Positive
100	10.9	Grid NO. 11	False	/	/	Positive
101	10.9	Grid NO. 11	True	95.63%	Yse	Positive
102	10.9	Grid NO. 11	True	100%	Yse	Positive
103	10.9	Grid NO. 11	False	/	/	Positive
104	10.9	Grid NO. 11	False	/	/	Positive
105	10.9	Grid NO. 11	True	98.3%	Yse	Positive
106	10.10	Grid NO. 12	False	/	/	/
107	10.10	Grid NO. 12	False	/	/	/
108	10.10	Grid NO. 12	False	/	/	/

109	10.10	Grid NO. 12	False	/	/	/
110	10.10	Grid NO. 12	False	/	/	/
111	10.10	Grid NO. 12	False	/	/	/
112	10.10	Grid NO. 12	False	/	/	/
113	10.10	Grid NO. 12	False	/	/	/
114	10.12	Grid NO. 13	False	/	/	/
115	10.12	Grid NO. 13	False	/	/	/
116	10.12	Grid NO. 13	False	/	/	/
117	10.12	Grid NO. 13	False	/	/	/
118	10.12	Grid NO. 13	False	/	/	/
119	10.12	Grid NO. 13	False	/	/	/
120	10.12	Grid NO. 13	False	/	/	/
121	10.12	Grid NO. 13	False	/	/	/
122	10.12	Grid NO. 13	False	/	/	/
123	10.12	Grid NO. 13	False	/	/	/
124	10.12	Grid NO. 13	False	/	/	/
125	10.12	Grid NO. 13	False	/	/	/
126	10.12	Grid NO. 13	False	/	/	/
127	10.12	Grid NO. 13	False	/	/	/
128	10.12	Grid NO. 13	False	/	/	/
129	10.12	Grid NO. 13	False	/	/	/
130	10.12	Grid NO. 13	False	/	/	/
131	10.12	Grid NO. 13	False	/	/	/
132	10.12	Grid NO. 13	False	/	/	/
133	10.12	Grid NO. 13	False	/	/	/
134	10.12	Grid NO. 13	False	/	/	/
135	10.12	Grid NO. 13	False	/	/	/
136	10.12	Grid NO. 13	False	/	/	/
137	10.14	Grid NO. 14	False	/	/	/
138	10.14	Grid NO. 14	False	/	/	/
139	10.14	Grid NO. 14	False	/	/	/
140	10.14	Grid NO. 14	False	/	/	/

141	10.14	Grid NO. 14	False	/	/	/
142	10.18	Grid NO. 15	False	/	/	/
143	10.18	Grid NO. 15	False	/	/	/
144	10.18	Grid NO. 15	False	/	/	/
145	10.18	Grid NO. 15	False	/	/	/
146	10.18	Grid NO. 15	False	/	/	/
147	10.22	Grid NO. 16	False	/	/	/
148	10.23	Grid NO. 16	False	/	/	/
149	10.23	Grid NO. 16	False	/	/	/
150	10.23	Grid NO. 16	False	/	/	/
151	10.23	Grid NO. 16	False	/	/	Positive
152	10.23	Grid NO. 16	False	/	/	/
153	10.23	Grid NO. 16	False	/	/	Positive
154	10.23	Grid NO. 16	False	/	/	/
155	10.23	Grid NO. 16	False	/	/	/
156	10.23	Grid NO. 16	True	97.41%	Yse	Positive
157	10.23	Grid NO. 16	False	/	/	/
158	10.23	Grid NO. 16	False	/	/	/
159	10.23	Grid NO. 16	False	/	/	Positive
160	10.23	Grid NO. 16	False	/	/	/
161	10.23	Grid NO. 16	False	/	/	/
162	10.23	Grid NO. 16	False	/	/	/
163	10.23	Grid NO. 16	False	/	/	/
164	10.23	Grid NO. 16	False	/	/	/
165	10.23	Grid NO. 16	False	/	/	/
166	10.23	Grid NO. 16	False	/	/	/
167	10.23	Grid NO. 16	False	/	/	/
168	10.23	Grid NO. 16	False	/	/	/
169	10.23	Grid NO. 16	False	/	/	/
170	10.25	Grid NO. 17	False	/	/	/
171	10.25	Grid NO. 17	False	/	/	/
172	10.25	Grid NO. 17	False	/	/	/

173	10.25	Grid NO. 17	False	/	/	/
174	10.25	Grid NO. 17	False	/	/	/
175	10.25	Grid NO. 17	False	/	/	/
176	10.25	Grid NO. 17	False	/	/	/
177	10.30	Grid NO. 18	False	/	/	/
178	10.30	Grid NO. 18	False	/	/	/
179	10.30	Grid NO. 18	False	/	/	/
180	10.30	Grid NO. 18	False	/	/	/
181	10.30	Grid NO. 18	False	/	/	/
182	10.30	Grid NO. 18	False	/	/	/
183	10.30	Grid NO. 18	False	/	/	/
184	10.30	Grid NO. 18	False	/	/	/
185	10.30	Grid NO. 18	False	/	/	/
186	10.30	Grid NO. 18	False	/	/	Positive
187	10.30	Grid NO. 18	False	/	/	/
188	10.30	Grid NO. 18	False	/	/	Positive
189	10.30	Grid NO. 18	False	/	/	Positive
190	10.30	Grid NO. 18	False	/	/	/
191	10.30	Grid NO. 18	False	/	/	/
192	10.30	Grid NO. 18	False	/	/	/
193	11.4	Grid NO. 19	False	/	/	/
194	11.4	Grid NO. 19	False	/	/	/
195	11.4	Grid NO. 19	False	/	/	/
196	11.4	Grid NO. 19	False	/	/	/
197	11.4	Grid NO. 19	False	/	/	/
